# Metabolic Syndrome and Autophagy: Focus on HMGB1 Protein

**DOI:** 10.3389/fcell.2021.654913

**Published:** 2021-04-12

**Authors:** Vincenza Frisardi, Carmela Matrone, Maria Elisabeth Street

**Affiliations:** ^1^Clinical and Nutritional Laboratory, Department of Geriatric and NeuroRehabilitation, Arcispedale Santa Maria Nuova (AUSL-IRCCS), Reggio Emilia, Italy; ^2^Division of Pharmacology, Department of Neuroscience, School of Medicine, University of Naples Federico II, Naples, Italy; ^3^Division of Paediatric Endocrinology and Diabetology, Paediatrics, Department of Mother and Child, Arcispedale Santa Maria Nuova (AUSL-IRCCS), Reggio Emilia, Italy

**Keywords:** metabolic syndrome, autophagy, HMBG1, cellular homeostasis, insulin resistance, oxidative stress

## Abstract

Metabolic syndrome (MetS) affects the population worldwide and results from several factors such as genetic background, environment and lifestyle. In recent years, an interplay among autophagy, metabolism, and metabolic disorders has become apparent. Defects in the autophagy machinery are associated with the dysfunction of many tissues/organs regulating metabolism. Metabolic hormones and nutrients regulate, in turn, the autophagy mechanism. Autophagy is a housekeeping stress-induced degradation process that ensures cellular homeostasis. High mobility group box 1 (HMGB1) is a highly conserved nuclear protein with a nuclear and extracellular role that functions as an extracellular signaling molecule under specific conditions. Several studies have shown that HMGB1 is a critical regulator of autophagy. This mini-review focuses on the involvement of HMGB1 protein in the interplay between autophagy and MetS, emphasizing its potential role as a promising biomarker candidate for the early stage of MetS or disease’s therapeutic target.

## Introduction

Metabolic syndrome (MetS) increases significantly morbidity and all-cause mortality worldwide (Cornier et al., 2008; Alamdari et al., 2020; Watanabe and Kotani, 2020). MetS is linked to cognitive decline and Alzheimer’s disease (AD), and this has suggested the term “metabolic-cognitive syndrome” (Frisardi et al., 2010). Over the past few decades, the prevalence of MetS, cardiovascular disease, and dementia has risen rapidly. The increasing worldwide prevalence of childhood obesity and diabetes in the young (DeBoer, 2019; Weihe and Weihrauch-Blûher, 2019) has promoted the search for biochemical markers of MetS to identify its prodromal phase or to predict the evolutionary risk. Autophagy is a degradation process facilitating homeostasis and intracellular energy balance. Emerging discoveries showed the complex and reciprocal interplay between autophagy and metabolism (Martinez-Lopez et al., 2017; Raj et al., 2020). Obesity, fatty liver disease and diabetes, the principal components of MetS, show dysregulated hepatic autophagy (Zhang et al., 2018; Allaire et al., 2019). Vice versa, glycolysis alters the autophagy self-fueling derangements in other metabolic pathways (Kiffin et al., 2006). A ubiquitous small chromatin-linked non-histone peptide, High Mobility Group Box-1 (HMGB1), has gained attention lately as a critical promoter of autophagy processes (Huebener et al., 2014). HMGB1 levels are related to inflammation (Cal et al., 2015), insulin resistance (IR), hyperglycemia (Migazzi et al., 2021), and MetS (Jialal et al., 2014; Chen et al., 2020a). Understanding the molecular bases for these processes is essential for developing new diagnostic biomarkers and identifying new therapeutic target and subpopulations at risk. Not all obese subjects have Mets, while lean subjects could develop MetS-linked cardiovascular complications.

## Metabolic Syndrome (MetS): Dysfunctional Adiposity and IR

Metabolic and vascular factors, especially visceral obesity and IR, characterize MetS (Cornier et al., 2008). Measuring IR is demanding, and the lack of assay standardization makes new syndrome markers necessary. Chronic pro-inflammatory and pro-thrombotic states, non-alcoholic fatty liver disease (NAFLD) (Lonardo et al., 2020), and sleep apnea (Castaneda et al., 2018) contribute to the MetS entity (Jabalie et al., 2019). MetS represents a clinical spectrum where a lag time exists between a single risk factor, the syndrome’s definition and clinical consequences. Receiving a diagnosis of MetS is already too late (Reaven, 2006) compared to the possibility of having molecular markers of disease’s evolving risk. Adipose tissue (AT) is metabolically active (Kershaw and Flier, 2004; Iozzo and Guzzardi, 2016). Chronic nutrient surplus and hyperinsulinemia increase adipocytes metabolic glucose flux and lead to cell hypertrophy. As adipocytes reach the critical size, precursor cells differentiate (Longo et al., 2019). In this context, lipogenic and antilipolytic control is impaired with reduced insulin sensitivity and ectopic fat accumulation. Therefore, the inability to buffer excess metabolic substrates from nutritional overload exposes other tissues to lipotoxicity (Cornier et al., 2011). There are functional differences between healthy (insulin sensitive) and unhealthy (IR) obesity. Inflammation can induce DNA damage, such as DNA double-strand breaks (DSBs), which increase inflammation. Obesity also modifies the immune cells (Olefsky and Glass, 2010; Trim et al., 2018). Explicitly, in the AT of obese subjects, monocytes polarize to M1 macrophages and display several cytokines (including TNF-α, IL-6, HMGB1) (Zhang et al., 2017). This molecular shift aggravates the chronic inflammatory state and IR. Furthermore, an increased formation of advanced glycation products (AGE) and their signaling via specific receptors (RAGE), including redox mechanisms, mediate vascular dysfunction and end-organ failure in MetS (Fournet et al., 2018).

## Overview on Autophagy

**FIGURE 1:**
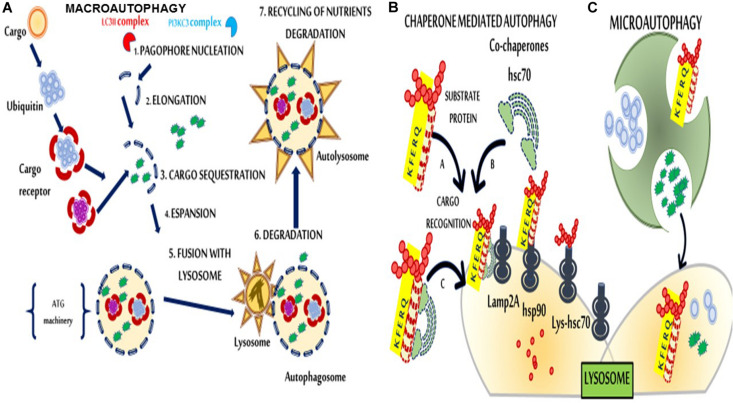
Schematic representation of macroautophagy, chaperone-mediated autophagy (CMA), and microautophagy. **(A)** In macroautophagy, cargos are sequestered by phagophores, which elongate and form a double membranous structure, the autophagosome. Autophagosomes then fuse with the lysosome to form autolysosomes. (1) Nucleation consists of the formation of the phagophore. A class III of phosphoinositide 3-kinases (PI3K) complex consisting of beclin 1 (BCN1), Phosphatidylinositol 3-kinase catalytic subunit type 3 (PIK3C3), Phosphoinositide 3-kinase regulatory subunit 4 (PIK3R4), UV radiation resistance-associated gene protein (UVRAG), and Autophagy And Beclin 1 Regulator 1 (AMBRA1) is required for phagophore formation. (2) Microtubule-associated proteins 1A/1B light chain 3B (MAP1LC3) complex anchors to the membrane via a phosphoethanolamine (PE) anchor (LC3-II) and triggers the elongation. (3) The phagophore sequesters cytosolic cargo and forms a double-membranous vesicle, the autophagosome. (4) Maturation, the completed autophagosome undergoes multiple maturation steps. (5) Docking and fusion, the autophagosome is released into the lysosome/autolysosome to be degraded by lysosomal hydrolases or to become available for re-usage (6). **(B)** In CMA **(left)**, substrate proteins that can be damaged by various factors, such as reactive oxygen species (ROS), bind the Lysosomial- Heat shock cognate 71 kDa protein (Lys-Hsc70) chaperone through a specific amino acid sequence (the KFERQ motif) and are transported across the lysosomal membrane for degradation via interaction with lysosomal-associated membrane protein 2A (Lamp2A) proteins. **(C)** Microautophagy **(right)** involves the direct engulfment of portions of the cytoplasm into lysosomes.

Although firstly described in 1963, only in the 1990s autophagy mechanisms were elucidated with identifying autophagy-related genes (ATG) in yeast (Takeshige et al., 1992; Klionsky, 2007; Mizushima, 2018). In eukaryotic, energy deprivation and/or intense physical activity trigger the cellular self-digestion processes to secure sufficient nutrient supply (Klionsky, 2007; Maiuri et al., 2007; Mizushima et al., 2008; Levine et al., 2011; Rubinsztein et al., 2011). Besides its role in preserving normal cellular functions, autophagy participates in several diseases (Jiang and Mizushima, 2014). Many stress conditions lead to a progressive accumulation of toxic molecular components and activate autophagic processes (Leidal et al., 2018) that rely upon three primary types: microautophagy, macroautophagy, and chaperone-mediated autophagy (CMA). Macroautophagy differs from the others because the waste of damaged organelles, unneeded cellular materials, and pathogenic agents are first sequestered and encapsulated in double-membrane vesicles (autophagosomes). Then, by trafficking from the cytoplasm to the lysosomes, autophagosomes fuse with lysosomes, and their contents can be either recycled or degraded (Mehrpour et al., 2010; Lamb et al., 2013; Figure 1). Six main steps (initiation, nucleation, elongation closure, maturation, and degradation or extrusion) characterize autophagy; each of these is highly regulated (Figure 1). Beclin 1 (BCN1) belongs to the autophagy machinery, and it plays its effects by the activation of specific (BCN1)-binding proteins, autophagic inducers and autophagic inhibitors in a cell- or tissue-dependent fashion (Cuomo et al., 2019). Autophagy induction might counteract SARS-CoV-2 infection (Carmona-Gutierrez et al., 2020). Although it is far beyond the goal of this review, the speculation of autophagy as a possible druggable target in SARS-CoV-2 is undeniably and surely deserves further investigations, also regarding the hypothesized link among obesity, IR and COVID-19 (Frisardi, 2020; Street, 2020).

## Autophagy and MetS: The Vicious Circle

**FIGURE 2:**
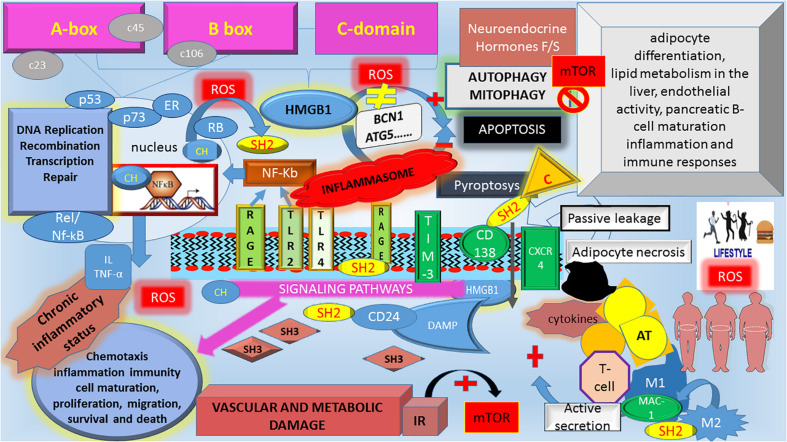
The schematic interplay among HMBG1, autophagy, and Metabolic Syndrome. HMGB1, High mobility group box 1; c23, c46, c106, cysteine at 23, 46, 106 position; ROS, reactive oxygen species; BCN1, Beclin-1; ATG, autophagy-related genes; Neuroendocrine hormones F/S, fasting/starvation; mTOR, mammalian target of rapamycin; NF-κb, nuclear factor kappa-light-chain-enhancer of activated B cells; IL, interleukines; TNF-α, tumor necrosis factor-alpha; p53, protein 53; p73, protein 73; RB, retinoblastoma protein; Rel/Nf-κB, member of Rel/Nf-κB family; ER, estrogen receptor; C, caspases, non-canonical inflammasome; RAGE, advanced glycosylation end product-specific receptor; TLR2 and TLR4, Toll-like Receptor 2 and 4; TIM-3, T-cell immunoglobulin mucin-3; CXCR4, chemokine C-X-C motif receptor 4; Mac-1, macrophage-1 antigen; CD138, syndecan-1; CD24, cluster of differentiation 24; M, macrophages; IR, Insulin Resistance; AT, Adipose Tissue. HMGB1 functions as a Damage Associated Molecular Pattern (DAMP) protein in the extracellular space. A mixture of different HMBG1 isoforms (CH = reduced form; SH2: disulfide HMBG1, SH3 oxidized form) in the extracellular space activates different pathway signaling. As the disulfide HMBG1 is responsible for autophagy activation, which counteracts the metabolic consequences of MetS. During an overload of food nutrients, there is an increase in ROS. The oxidative environment modifies the Reduced/oxidized HMBG1 ratio, increasing the dysregulation in Insulin/mTOR signaling, which blocks the autophagy machinery. It follows an increased risk for the “unhealthy” obese to develop MetS complications due to an unbalance among the downstream IR pathway, chronic inflammatory pattern and inability to counteract metabolic derangements via autophagy machinery disruption. HMGB1 is an autophagic regulator that mediates stress response: in normal condition, cytosolic HMGB1 as BECN1-binding protein induces autophagy. Extracellular HMGB1 binds RAGE, which inhibits mTOR and promotes autophagy. In chronic obesity and switching from insulin-sensitive to IR state, change in oxidative environment modifies the HMBG1 activity altering its inducer role in autophagy. In the nucleus, HMBG1 interact with and enhances the activities of number of transcription factors, including p53, p73, RB, Rel/Nf-κB, and ER. Once released, HMBG1 binds to various receptors to activate DAMP signaling involved in multiple cellular processes. Inflammasomes are a cytosolic multiprotein complex formation that are recruited by external pathogen and/or internal stimuli. Chronic inflammasomes lead to chronic inflammatory status increasing the risk of clinical consequences of MetS. HMGB1 triggers C (Caspase-4/caspase-5) which are components of the “non-canonical inflammasome” with cytokines release and induction of pyroptosis (a kind of proinflammatory cell death combining features of both apoptosis and necrosis).

Various metabolic disorders showed functional defects in autophagy (Ichimura and Komatsu, 2011; Ueno and Komatsu, 2017; Barbosa et al., 2018; Ren et al., 2018; Zhang et al., 2018). Over the last years, the use of mice models, yeast screen and genome-wide analysis has considerably amplified our knowledge about this topic (Li et al., 2016; Kuma et al., 2017). Silencing of ATG promotes obesity and triggers metabolic complications. Consistently, ATG overexpression improves the metabolic profile in aged mice (Pyo et al., 2013). Since fasting activates autophagy, dietary interventions promoting autophagy has been explored (Martinez-Lopez et al., 2017). Different metabolic phenotypes have been described in various tissues, suggesting that autophagy genes are differentially expressed and activated in a tissue- and stage-specific manner during the development. However, it is worth to note that deficits in the autophagy genes, at systemic rather than tissue-specific level, affect cell adaptation to metabolic stress more and facilitates the progression from a risk factor (ex. obesity) to full-blown diseases (Lim et al., 2014; Ren et al., 2018). Nutrient limitation and multiple stress conditions upregulate autophagy because this latter serves cytoprotective functions and, reducing cellular death, limits the following inflammatory state. Indeed, autophagy could represent a protective mechanism following myocardial infarction (Czaja, 2010). Autophagy regulates adipocyte differentiation, lipid metabolism, endothelial activity, pancreatic β-cell maturation, molecular processes related to inflammation/immune responses (Ryter et al., 2014) and storing of lipids. Whenever autophagy is inhibited, lipids accumulate, and many processes’ dysregulation occurs (Czaja, 2010). Briefly, cell-intrinsic effects (e.g., nutrient metabolism, mitochondria, and lipid droplet homeostasis), cell-extrinsic effects (e.g., the release of pro-inflammatory cytokines), and potentially lack of feedback inhibition of insulin and mTOR-C1 (mammalian target of rapamycin-complex 1) signaling pathways interfere with autophagy mechanisms. As in a vicious circle, defects in autophagy accelerates lifestyle-induced obesity that, in turn, inhibits autophagy in the liver, muscle and AT, worsening symptomatic features of MetS (Figure 2; Yang et al., 2010; Ruderman et al., 2013; Kaur and Debnath, 2015; Ren and Xu, 2015; Che et al., 2018; Zhang et al., 2018; Menikdiwela et al., 2020). In obesity, autophagy is suppressed via an increase in mTOR activity, which is involved in different cardiovascular pathophysiology (Wang et al., 2007; Che et al., 2018; Samidurai et al., 2018). Chronic obesity related-stress with a dysregulation of insulin/mTOR signaling (Wang et al., 2007; Sohrabi et al., 2019; Menikdiwela et al., 2020) lead to autophagy machinery disruption (Figure 2). During fasting, neuroendocrine signals [e.g., insulin and Insulin Growth Factor 1 decrease vs. glucagon, fibroblast growth factor 21 (FGF21) increase] regulate autophagy tightly. Commonly, these neurohormonal signals are altered in obesity (Levine and Kroemer, 2019). Mesenchymal stem cells from patients with diabetes and MetS show changes in oxidative stress and autophagy (Kornicka et al., 2018). Both lipogenesis and adipogenesis are redox-sensitive; healthy obesity is consistent with the lack of the redox stress signature (Jankovic et al., 2015; Bañuls et al., 2017; Böhm et al., 2020). On the contrary, constant nutritional overload and oxidative pressure may compromise autophagy machinery ability to counteract metabolic derangement. As oxidative injuries accumulate, irreversible damage appears with signaling pathways disruption. Consistently, data suggest that oxidative injury may precede adipocyte dysfunction and other metabolic disorders even in yet metabolically healthy obese subjects (Jankovic et al., 2015). Evidence support an association between mitochondrial dysfunction and MetS in prediabetic and diabetic states (Bugger and Abel, 2008; Montgomery, 2019; Böhm et al., 2020). Mitophagy (selective autophagy in mitochondria) is a vital mechanism to keep stable the metabolic homeostasis (Xu et al., 2020). Therefore, enhancement of autophagy activity might be a novel therapeutic approach against organ failure’s evolving metabolic disorders.

## HMGB1: A Multifaceted Protein

High mobility group box 1 (HMGB1) is an evolutionarily highly conserved small chromatin-linked non-histone peptide, first identified in the HMG family. HMGB1 (215 amino acids) is organized in three distinct regions: Box A and Box B and the C-terminal domain. While Box A and B are essential for the HMGB1 binding to DNA and thereby regulating transcription of target genes, the C-terminal domain contains the binding sites for RAGE and Toll-like receptor (TLR) (Livesey et al., 2012; Jiang et al., 2020). Each of these receptors mediates HMGB1 signals (Park et al., 2004), also activating the NF-κB proinflammatory pathway (nuclear factor kappa-light-chain-enhancer of activated B cells) (Jiang et al., 2020; Figure 2). NF-κB was also detected in the mitochondria, where it intervenes in mitochondrial dynamics, apoptosis, respiratory control, gene expression, and disease mechanisms (Albensi, 2019). HMGB1 is involved in maintaining genomic structure and function, and it is predominantly located in the nucleus in the reduced form Tang et al. (2010a). However, a small amount of HMBG1 is also present in the cytosol, which controls cell stress responses and inhibits apoptosis (Yang et al., 2013). During inflammation, HMGB1 promotes autophagy via binding to BCN1 and ATG5 and regulating mitochondrial morphology and function (Zhu et al., 2015; Figure 2).

HMBG1 can be released either passively from damaged cells or actively from immunologically activated immune cells under distress conditions. Extracellular HMGB1 acts as an alarmin and a Damage Associated Molecular Pattern (DAMP) protein (Raucci et al., 2019) by binding to several pathogen-associated molecular patterns (PAMPs) (Jiang et al., 2020) and activating downstream signals (Figure 2). An excessive accumulation of extracellular HMGB1 has been associated with the pathogenesis of many disorders, including diabetes (Wang et al., 2016; Zhang et al., 2020; Li and Lu, 2021). HMGB1 has several extracellular receptors (Figure 2). Actually, only RAGE and TLR4 are mainly studied and reported receptors (Andersson et al., 2018). HMGB1 serves as a redox sensor. In this regard, the three conserved redox-sensitive cysteine residues Cys23, Cys45, and Cys106 play a critical role (Li et al., 2003). Depending on the redox state, HMGB1 switches from the active to the inactive conformation. In particular, when Cys106 is oxidized, HMGB1 is inactive and likely promotes immune tolerance with the release of proinflammatory cytokines. Moreover, Cys106 oxidation induces the HMGB1 dimerization in cells exposed to oxidative stress. It follows that HMGB1 binds to DNA with a higher affinity than monomeric HMGB1, protects DNA from damage due to hydroxyl free radicals and prevents cell death (Kwak et al., 2021).

Conversely, the reduced HMGB1 form switches its activity into the proinflammatory state (Zhang et al., 2017). Although the oxidized HMGB1 is thought to be non-inflammatory, a role in promoting the intrinsic apoptotic pathway has been reported (Tang et al., 2010b). Moreover, a mixture of oxidized/reduced HMGB1 isoforms has been described in the extracellular compartments exerting different effects on cell defense mechanisms (Xue et al., 2020).

## HMGB1, Autophagy and MetS: A Suggestive Triangulation

Accumulating evidence supports the relationship between autophagy and HMGB1. There is a mutual regulation where one’s inhibition affects the release of the other (Zhang et al., 2017), while uncontrolled autophagy increases the HMGB1 release (Tang et al., 2010b; Kim et al., 2020). HMGB1 is crucial for normal autophagy functioning (Tang et al., 2010a; Foglio et al., 2019). As a transcriptional co-factor, HMGB1 regulates the expression of heat shock protein β-1 (Tang et al., 2011; Foglio et al., 2019), which sustains dynamic intracellular trafficking during autophagy. Cytosolic HMGB1 competes with Bcl-2 for interaction with BCN1 by intramolecular disulfide bridge of HMGB1 promoting BCN1-mediated autophagosomes (Kang et al., 2010; Tang et al., 2010a; Foglio et al., 2019). HMGB1 triggers autophagy through binding to RAGE (Tang et al., 2010a). This latter is a positive regulator of autophagy and a negative regulator of apoptosis during oxidative stress, DNA damage, and hypoxia (Kang et al., 2010). In *in vitro* and *in vivo* experiments, deletion, depletion or inhibition of HMGB1 reduces autophagy (Tang et al., 2010a, b). HMGB1-mediated autophagy prevents a worse evolution of several diseases (Kang et al., 2014). In contrast, conditional knockdown of HMGB1 in the liver or heart does not affect autophagy and mitochondrial quality (Huebener et al., 2014). These conflicting results could be imputable first to the difference in cellular line; second, we can hypothesize that as a DAMP, over secreted HMBG1 could play its role in a paracrine mode by linking RAGE in the target organ. In the Huebener et al. (2014) experiment, HMGB1 was deleted in hepatocytes but not in non-parenchymal liver cells. RAGE expression was only found on ductal cells and Kupffer’s cells but not on hepatocytes and this could the explanation of why in the experiment performed by Huebener et al. (2014) deleted HMBG1 in hepatocytes does not alter mitophagy, autophagy, or gene expression.

Furthermore, in basal condition, maybe HMBG1 could be dispensable for autophagy. Nevertheless, under stress conditions, if we modify the cellular micro-macro environment, for example, by aging, cumulating oxidative damage or nutrients overload, HMBG1 could be essential (Ferrara et al., 2020). In atherosclerotic lesions in human carotid, BCN1 was found to co-localize with HMGB1 and were both found in foamy macrophages suggesting an interplay between HMGB1 and autophagy in atherosclerosis (Umahara et al., 2020). Further studies are re required to investigate the HMGB1 contributes to autophagy in tissue-specific contexts and conditions.

Differently than in the inflammation (Cal et al., 2015; Yao et al., 2015; Biscetti et al., 2019) and autophagy, the role exerted by HMGB1 in MetS and its potential contribution to cardiovascular complications remains mostly unexplored despite the increasing number of evidence underlining this association (van Niekerk et al., 2019). A linear relationship has been consistently observed among HMGB1 levels and inflammation, IR, and hyperglycemia (Montanini et al., 2016; Cirillo et al., 2019, 2020). In particular, a study comparing control mice to MetS mice, fed with a high-fat diet, showed increased secretion of HMGB1 in the AT of the affected mice (Jialal et al., 2014). Increased circulating HMGB1 concentrations have been described in obese children with MetS compared to healthy controls (Arrigo et al., 2013).

Further, in adipocytes, HMGB1 secretion is regulated by c-Jun (Shimizu et al., 2016), a downstream mediator of the insulin receptor. HMGB1 is implicated in the development of non-alcoholic fatty liver Disease (NAFLD) by insulin receptor downstream effectors (Arrigo et al., 2013; Wang et al., 2015; Giacobbe et al., 2016). Obese pregnant women as children show high serum HMGB1 levels (Arrigo et al., 2013; Giacobbe et al., 2016), directly associated with body mass index. Circulating HMGB1 significantly increase in obese individuals and T2D patients (Wang et al., 2015). However, a larger sample size will be necessary to support the clinical relevance of HMGB1 as a potential and viable biomarker for the early diagnosis of obesity. As secreted by the macrophages within AT (Bonaldi et al., 2003), HMGB1 may promote inflammation by binding to receptors on effector cell membranes, leading to inflammatory mediators (IL-6 and TNF-α). In turn, the release of IL-6 and TNF-α leads to increased HMGB1 release, resulting in a cascade amplification of inflammation (Zhang et al., 2019).

High mobility group box 1-gene-deficient mice show several metabolic defects and die of hypoglycemia. Obese individuals are more prone to DNA damage than normal-weight adolescents (Azzarà et al., 2016; Rohde et al., 2020) but have improved the potential to repair occurred lesions. Different repair kinetics of DSBs in obese versus lean derived lymphocytes, along with differences in HMGB1 expression level, have been reported, and specifically, cytoplasmic HMGB1 is more abundant in VAT (visceral adipose tissue) of obese compared with lean subjects (Azzarà et al., 2016). To find early biomarkers of autophagy/apoptosis unbalance concerning MetS principally, HMGB1 could represent a seducing molecule (Foglio et al., 2019). Although most evidence came from studies on cancer cell lines, speculation could be made as the cancer cells are exposed to a metabolically demanding environment (Marijt et al., 2019). Conjectures concerning a pivotal role for HMBG1 could also derive from the observed increased risk among obese subjects in morbidity and mortality related to COVID-19 (Seidu et al., 2020). Shortly, HMGB1 is (1) related to an increased risk of thrombosis; (2) HMGB1 gene polymorphisms are associated with hypertension; (3) HMGB1 regulates ACE II receptors which act as a counterbalance to the Angiotensin-converting enzyme (ACE), the central component of the renin-angiotensin system (Chen et al., 2020b) essential for SARS-CoV-2 infection (Chen et al., 2020b; Street, 2020; Figure 1B). *In vitro* studies have shown that in bronchial epithelial cells, hyperglycemia increases HMGB1 while it is lowered by insulin (Montanini et al., 2016; Seidu et al., 2020), suggesting that this protein might be a vulnerability marker besides a therapeutic target.

## Conclusion

This mini-review focused on the hypothetical involvement of HMGB1 in the current hot topic of autophagy and MetS to prompt debate and promote further experimental studies. Chronic nutrient overload impairs the autophagy mechanism’s ability to counteract the lifestyle-induced metabolic processes, and it appears that autophagy defects play a role in determining the cardiovascular complications of MetS. HMGB1, among many other functions, also regulates autophagy and therefore represents an attractive biomarker of disease evolution and a possible therapeutic target. Obese subjects have elevated serum levels of HMGB1. We underlined the possible importance of the reducing/oxidized HMGB1 ratio for predicting the risk of disease evolution in obese healthy subjects using a conceptual “autophagy bridge.” Early diagnosis of a metabolic state that will progress to MetS complications is of crucial importance.

## Author Contributions

VF conceived of the presented idea and planned the manuscript. CM conceived Figure 1 and VF conceived of Figure 2. All authors shared the leading role in writing the manuscript, read and agreed to the published version of the manuscript.

## Conflict of Interest

The authors declare that the research was conducted in the absence of any commercial or financial relationships that could be construed as a potential conflict of interest.

## References

[B1] Alamdari N. M. Rahimi F. S. Afaghi S. Zarghi A. Qaderi S. Tarki F. E. (2020). The impact of metabolic syndrome on morbidity and mortality among intensive care unit admitted COVID-19 patients. *Diabetes Metab. Syndr.:Clin. Res. Rev.* 14 1979–1986. 10.1016/j.dsx.2020.10.012 33080538 PMC7550894

[B2] Albensi B. C. (2019). What Is Nuclear Factor Kappa B (NF-κB) Doing in and to the Mitochondrion? *Front. Cell Dev. Biol.* 2019:00154. 10.3389/fcell.2019.00154 31448275 PMC6692429

[B3] Allaire M. Rautou P. E. Codogno P. Lotersztajn S. (2019). Autophagy in liver diseases: time for translation? *J. Hepatol.* 70 985–998. 10.1016/j.jhep.2019.01.026 30711404

[B4] Andersson U. Yang H. Harris H. (2018). High-mobility group box 1 protein (HMGB1) operates as an alarmin outside as well as inside cells. *Semin Immunol.* 38 40–48. 10.1016/j.smim.2018.02.011 29530410

[B5] Arrigo T. Chirico V. Salpietro V. Munafò C. Ferraù V. Gitto E. (2013). High-mobility group protein B1: a new biomarker of metabolic syndrome in obese children. *Eur. J. Endocrinol.* 68 631–638. 10.1530/EJE-13-0037 23384711

[B6] Azzarà A. Pirillo C. Giovannini C. Federico G. Scarpato R. (2016). Different repair kinetic of DSBs induced by mitomycin C in peripheral lymphocytes of obese and normal weight adolescents. *Mutat Res.* 789 9–14. 10.1016/j.mrfmmm.2016.05.001 27174706

[B7] Bañuls C. Rovira-Llopis S. Lopez-Domenech S. Diaz-Morales N. Blas-Garcia A. Veses S. (2017). Oxidative and endoplasmic reticulum stress is impaired in leukocytes from metabolically unhealthy vs healthy obese individuals. *Int. J. Obes.* 41 1556–1563. 10.1038/ijo.2017.147 28630460

[B8] Barbosa M. C. Grosso R. A. Fader C. M. (2018). Hallmarks of Aging: An Autophagic Perspective. *Front. Endocrinol.* 9:790. 10.3389/fendo.2018.00790 30687233 PMC6333684

[B9] Biscetti F. Rando M. M. Nardella E. Cecchini A. L. Pecorini G. Landolfi R. (2019). High mobility group box-1 and diabetes mellitus complications: state of the art and future perspectives. *Int. J. Mol. Sci.* 20:E6258. 10.3390/ijms20246258 PMC6940913 31835864

[B10] Böhm A. Keuper M. Meile T. Zdichavsky M. Fritsche A. Häring H. U. (2020). Increased mitochondrial respiration of adipocytes from metabolically unhealthy obese compared to healthy obese individuals. *Sci. Rep.* 10:12407. 10.1038/s41598-020-69016-9 32709986 PMC7382448

[B11] Bonaldi T. Talamo F. Scaffidi P. Ferrera D. Porto A. Bachi A. (2003). Monocytic cells hyperacetylate chromatin protein HMGB1 to redirect it towards secretion. *EMBO J.* 22 5551–5560. 10.1093/emboj/cdg516 14532127 PMC213771

[B12] Bugger H. Abel E. D. (2008). Molecular mechanisms for myocardial mitochondrial dysfunction in the metabolic syndrome. *Clin. Sci.* 114 195–210. 10.1042/CS20070166 18184113

[B13] Cal J. Yuan H. Wang Q. Yang H. Al-Abed Y. Hua Z. (2015). HMGB1-driven inflammation and intimal hyperplasia after arterial injury involves cell-specific actions mediated by TLR4. *Arterioscler. Thromb. Vasc. Biol.* 35 2579–2593. 10.1161/ATVBAHA.115.305789 26515416 PMC4880018

[B14] Carmona-Gutierrez D. Bauer M. A. Zimmermann A. Kainz K. Hofer S. J. Kroemer G. (2020). Digesting the crisis: autophagy and coronaviruses. *Microb. Cell* 7 119–128. 10.15698/mic2020.05.715 32391393 PMC7199282

[B15] Castaneda A. Jauregui-Maldonado E. Ratnani I. Varon J. Surani S. (2018). Correlation between metabolic syndrome and sleep apnea. *World J. Diabetes* 9 66–71. 10.4239/wjd.v9.i4.66 29765510 PMC5951892

[B16] Che Y. Wang Z. Yuan Y. Zhang N. Jin Y. Wan C. (2018). Role of autophagy in a model of obesity: A long-term high fat diet induces cardiac dysfunction. *Mole. Med. Rep.* 18 3251–3261. 10.3892/mmr.2018.9301 30066870 PMC6102660

[B17] Chen L. Long X. Xu Q. Tan J. Wang G. Cao Y. (2020b). Elevated serum levels of S100A8/A9 and HMGB1 at hospital admission are correlated with inferior clinical outcomes in COVID-19 patients. *Cell Mol. Immunol.* 17 992–994. 10.1038/s41423-020-0492-x 32620787 PMC7332851

[B18] Chen L. Zhu H. Su S. Harshfield G. Sullivan J. Webb C. (2020a). High-Mobility Group Box-1 Is Associated With Obesity, Inflammation, and Subclinical Cardiovascular Risk Among Young Adults. *Arterioscler. Thromb. Vasc. Biol.* 2020:314599. 10.1161/atvbaha.120.314599 32814439 PMC7578115

[B19] Cirillo F. Catellani C. Lazzeroni P. Sartori C. Nicoli A. Amarri S. (2019). MiRNAs Regulating Insulin Sensitivity Are Dysregulated in Polycystic Ovary Syndrome (PCOS) Ovaries and Are Associated With Markers of Inflammation and Insulin Sensitivity. *Front. Endocrinol.* 13:879. 10.3389/fendo.2019.00879 31920988 PMC6923204

[B20] Cirillo F. Catellani C. Lazzeroni P. Sartori C. Tridenti G. Vezzani C. (2020). HMGB1 is increased in adolescents with polycystic ovary syndrome (PCOS) and decreases after treatment with myo-inositol (MYO) in combination with alpha-lipoic acid (ALA). *Gynecol. Endocrinol.* 36 588–593. 10.1080/09513590.2020.1725967 32054355

[B21] Cornier M. A. Dabelea D. Hernandez T. L. Lindstrom R. C. Steig A. J. Stob N. R. (2008). The metabolic syndrome. *Endocr. Rev.* 29 777–822. 10.1210/er.2008-0024 18971485 PMC5393149

[B22] Cornier M. A. Després J. P. Davis N. Grossniklaus D. A. Klein S. Lamarche B. (2011). Assessing adiposity: A scientific statement from the American Heart Association. *Circulation* 124 1996–2019. 10.1161/CIR.0b013e318233bc6 21947291

[B23] Cuomo F. Altucci L. Cobellis G. (2019). Autophagy Function and Dysfunction: Potential Drugs as Anti-Cancer Therapy. *Cancers* 11:1465. 10.3390/cancers11101465 31569540 PMC6826381

[B24] Czaja M. J. (2010). Autophagy in health and disease. 2. Regulation of lipid metabolism and storage by autophagy: pathophysiological implications. *Am. J. Physiol. Cell Physiol.* 298 C973–C978. 10.1152/ajpcell.00527.2009 20089934 PMC2867392

[B25] DeBoer M. D. (2019). Assessing and Managing the Metabolic Syndrome in Children and Adolescents. *Nutrients* 11:1788. 10.3390/nu11081788 31382417 PMC6723651

[B26] Ferrara M. Chialli G. Ferreira L. M. Ruggieri E. Careccia G. Preti A. (2020). Oxidation of HMGB1 Is a Dynamically Regulated Process in Physiological and Pathological Conditions. *Front. Immunol.* 11:1122. 10.3389/fimmu.2020.01122 32670275 PMC7326777

[B27] Foglio E. Pellegrini L. Germani A. Russo M. A. Limana F. (2019). HMGB1-mediated apoptosis and autophagy in ischemic heart diseases. *Vasc. Biol.* 1 H89–H96. 10.1530/VB-19-0013 32923959 PMC7439920

[B28] Fournet M. Bonté F. Desmoulière A. (2018). Glycation Damage: A Possible Hub for Major Pathophysiological Disorders and Aging. *Aging Dis.* 9 880–900. 10.14336/AD.2017.1121 30271665 PMC6147582

[B29] Frisardi V. (2020). Commentary: Coronavirus and Obesity: Could Insulin Resistance Mediate the Severity of Covid-19 Infection? *Front. Public Health* 8:351. 10.3389/fpubh.2020.00351 32733840 PMC7358339

[B30] Frisardi V. Solfrizzi V. Seripa D. Capurso C. Santamato A. Sancarlo D. (2010). Metabolic-cognitive syndrome: a cross-talk between metabolic syndrome and Alzheimer’s disease. *Ageing Res. Rev.* 9 399–417. 10.1016/j.arr.2010.04.007 20444434

[B31] Giacobbe A. Granese R. Grasso R. Salpietro V. Corrado F. Giorgianni G. (2016). Association between maternal serum high mobility group box 1 levels and pregnancy complicated by gestational diabetes mellitus. *Nutr. Metab. Cardiovasc.* 26 414–418. 10.1016/j.numecd.2016.02.007 27089978

[B32] Huebener P. Gwak G. Y. Pradere J. P. Quinzii C. M. Friedman R. Lin C. S. (2014). High Mobility Group Box 1 is Dispensable for Autophagy, Mitochondrial Quality Control and Organ Function in Vivo. *Cell Metab.* 19 539–547. 10.1016/j.cmet.2014.01.014 24606906 PMC4099361

[B33] Ichimura Y. Komatsu M. (2011). Pathophysiological role of autophagy: lesson from autophagy-deficient mouse models. *Exp. Anim.* 60 329–345. 10.1538/expanim.60.329 21791873

[B34] Iozzo P. Guzzardi M. A. (2016). Cross-Talk Between Adipose Tissue Health, Myocardial Metabolism and Vascular Function: The Adipose-Myocardial and Adipose-Vascular Axes. *Curr. Pharm. Des.* 22 59–67. 10.2174/1381612822666151109111834 26548309

[B35] Jabalie G. Ahmadi M. Koushaeian L. Eghbal-Fard S. Mehdizadeh A. Kamrani A. (2019). Metabolic syndrome mediates proinflammatory responses of inflammatory cells in preeclampsia. *Am. J. Reprod Immunol.* 81:e13086. 10.1111/aji.13086 30614120

[B36] Jankovic A. Korac A. Buzadzic B. Otasevic V. Stancic A. Daiber A. (2015). Redox implications in adipose tissue (dys)function–A new look at old acquaintances. *Redox Biol.* 6 19–32. 10.1016/j.redox.2015.06.018 26177468 PMC4511633

[B37] Jialal I. Rajamani U. Adams-Huet B. Kaur H. (2014). Circulating pathogen-associated molecular pattern - binding proteins and High Mobility Group Box protein 1 in nascent metabolic syndrome: implications for cellular Toll-like receptor activity. *Atherosclerosis* 236 182–187. 10.1016/j.atherosclerosis.2014.06.022 25063948

[B38] Jiang L. Shao Y. Tian Y. Ouyang C. Wang X. (2020). Nuclear Alarmin Cytokines in Inflammation. *J. Immunol. Res.* 4:7206451. 10.1155/2020/7206451 PMC7732391 33344656

[B39] Jiang P. Mizushima N. (2014). Autophagy and human diseases. *Cell Res.* 24 69–79. 10.1038/cr.2013.161 24323045 PMC3879707

[B40] Kang R. Chen R. Zhang Q. Hou W. Wu S. Cao L. (2014). HMGB1 in health and disease. (2014). *Mol. Aspects Med.* 40 1–116. 10.1016/j.mam.2014.05.001 25010388 PMC4254084

[B41] Kang R. Livesey K. M. Zeh H. J. Loze M. T. Tang D. (2010). HMGB1: a novel Beclin 1-binding protein active in autophagy. *Autophagy* 6 1209–1211. 10.4161/auto.6.8.13651 20935509

[B42] Kaur J. Debnath J. (2015). Autophagy at the crossroads of catabolism and anabolism. *Nat. Rev. Mol. Cell Biol.* 16 461–472. 10.1038/nrm4024 26177004

[B43] Kershaw E. E. Flier J. S. (2004). Adipose tissue as an endocrine organ. *J. Clin. Endocrinol. Metab.* 89 2548–2556. 10.1210/jc.2004-0395 15181022

[B44] Kiffin R. Bandyopadhyay U. Cuervo A. M. (2006). Oxidative stress and autophagy. *Antioxid Redox Signal.* 8 152–162. 10.1089/ars.2006.8.152 16487049

[B45] Kim Y. H. Kwak M. S. Lee B. Shin J. M. Aum S. Park I. H. (2020). Secretory autophagy machinery and vesicular trafficking are involved in HMGB1 secretion. *Autophagy* 2020:1826690. 10.1080/15548627.2020.1826690 33017561 PMC8496717

[B46] Klionsky D. J. (2007). Autophagy: from phenomenology to molecular understanding in less than a decade. *Nat. Rev. Mole. Cell Biol.* 8 931–937. 10.1038/nrm2245 17712358

[B47] Kornicka K. Houston J. Marycz K. (2018). Dysfunction of Mesenchymal Stem Cells Isolated from Metabolic Syndrome and Type 2 Diabetic Patients as Result of Oxidative Stress and Autophagy may Limit Their Potential Therapeutic Use. *Stem Cell Rev. Rep.* 14 337–345. 10.1007/s12015-018-9809-x 29611042 PMC5960487

[B48] Kuma A. Komatsu M. Mizushima N. (2017). Autophagy-monitoring and autophagy-deficient mice. *Autophagy* 13 1619–1628. 10.1080/15548627.2017.1343770 28820286 PMC5640176

[B49] Kwak M. S. Rhee W. J. Lee Y. J. Kim H. S. Kim Y. H. Kwon M. K. (2021). Reactive oxygen species induce Cys106-mediated anti-parallel HMGB1 dimerization that protects against DNA damage. *Redox Biol.* 40:101858. 10.1016/j.redox.2021.101858 33461096 PMC7815493

[B50] Lamb C. A. Yoshimori T. Tooze S. A. (2013). The autophagosome: origins unknown, biogenesis complex. *Nat. Rev. Mol. Cell Biol.* 14 759–774. 10.1038/nrm3696 24201109

[B51] Leidal A. M. Levine B. Debnath J. (2018). Autophagy and the cell biology of age-related disease. *Nat. Cell Biol.* 20 1338–1348. 10.1038/s41556-018-0235-8 30482941

[B52] Levine B. Kroemer G. (2019). Biological Functions of Autophagy Genes: A Disease Perspective Cell. *Volume* 176 11–42. 10.1016/j.cell.2018.09.048 PMC6347410 30633901

[B53] Levine B. Mizushima N. Virgin H. W. (2011). Autophagy in immunity and inflammation. *Nature* 469 323–335. 10.1038/nature09782 21248839 PMC3131688

[B54] Li J. Kokkola R. Tabibzadeh S. Yang R. Ochani M. Qiang X. (2003). Structural basis for the proinflammatory cytokine activity of high mobility group box 1. *Mol. Med.* 9 37–45. 12765338 PMC1430376

[B55] Li L. Lu Y. Q. (2021). The Regulatory Role of High-Mobility Group Protein 1 in Sepsis-Related Immunity. *Front. Immunol.* 22:601815. 10.3389/fimmu.2020 PMC7862754 33552058

[B56] Li W. Chen M. Wang E. Hu L. Hawkesford M. J. Zhong L. (2016). Genome-wide analysis of autophagy-associated genes in foxtail millet (Setaria italica L.) and characterization of the function of SiATG8a in conferring tolerance to nitrogen starvation in rice. *BMC Genomics.* 17:797. 10.1186/s12864-016-3113-4 27733118 PMC5062844

[B57] Lim Y. M. Lim H. Hur K. Y. Quan W. Lee H. Y. Cheon H. (2014). Systemic autophagy insufficiency compromises adaptation to metabolic stress and facilitates progression from obesity to diabetes. *Nat. Commun.* 5:4934. 10.1038/ncomms5934 25255859

[B58] Livesey K. M. Kang R. Vernon P. Buchser W. Loughran P. Watkins S. C. (2012). p53/HMGB1 complexes regulate autophagy and apoptosis. *Cancer Res.* 72 1996–2005. 10.1158/0008-5472 22345153 PMC3417120

[B59] Lonardo A. Leoni S. Alswat K. A. Fouad Y. (2020). History of Nonalcoholic Fatty Liver Disease. *Int. J. Mol. Sci.* 21:5888. 10.3390/ijms21165888 32824337 PMC7460697

[B60] Longo M. Zatterale F. Naderi J. Parrillo L. Formisano P. Raciti G. A. (2019). Adipose Tissue Dysfunction as Determinant of Obesity-Associated Metabolic Complications. *Int. J. Mol. Sci.* 20:2358. 10.3390/ijms20092358 31085992 PMC6539070

[B61] Maiuri M. C. Zalckvar E. Kimchi A. Kroemer G. (2007). Self-eating and self-killing: crosstalk between autophagy and apoptosis. *Nat. Rev. Mol. Cell Biol.* 8 741–752. 10.1038/nrm2239 17717517

[B62] Marijt K. A. Sluijter M. Blijleven L. Tolmeijer S. H. Scheeren F. A. van der Burg S. H. (2019). Metabolic stress in cancer cells induces immune escape through a PI3K-dependent blockade of IFNγ receptor signaling. *J. Immunother. Cancer* 7:152. 10.1186/s40425-019-0627-8 31196219 PMC6567539

[B63] Martinez-Lopez N. Tarabra E. Toledo M. Schwartz G. J. Kersten S. Singh R. (2017). System-wide Benefits of Intermeal Fasting by Autophagy. *Cell Metab.* 26 856.e–871.e. 10.1016/j.cmet.2017.09.020 29107505 PMC5718973

[B64] Mehrpour M. Esclatine A. Beau I. Codogno P. (2010). Autophagy in health and disease. Regulation and significance of autophagy: an overview. *Am. J. Physiol. Cell Physiol.* 298 C776–C785. 10.1152/ajpcell.00507.2009 20089931

[B65] Menikdiwela K. R. Ramalingam L. Rash F. Wang S. Dufour J. M. Kalupahana N. S. (2020). Autophagy in metabolic syndrome: breaking the wheel by targeting the renin-angiotensin system. *Cell Death Dis.* 11:87. 10.1038/s41419-020-2275-9 32015340 PMC6997396

[B66] Migazzi M. Dauriz M. Cirillo F. Catellani C. Villani M. Tosi F. (2021). Circulating HMGB1 Levels Are Associated With Glucose Clamp-Derived Measures of Insulin Resistance in Women With PCOS. *Accept. Endocr. Soc. Meeting^∗^* 2021. 10.1007/s40618-023-02119-y PMC10632283 37256493

[B67] Mizushima N. (2018). A brief history of autophagy from cell biology to physiology and disease. *Nat. Cell Biol.* 20 521–527. 10.1038/s41556-018-0092-5 29686264

[B68] Mizushima N. Levine B. Cuervo A. M. Klionsky D. J. (2008). Autophagy fights disease through cellular self-digestion. *Nature* 451 1069–1075. 10.1038/nature06639 18305538 PMC2670399

[B69] Montanini L. Cirillo F. Smerieri A. Pisi G. Giardino I. d’Apolito M. (2016). HMGB1 Is Increased by CFTR Loss of Function, Is Lowered by Insulin, and Increases In Vivo at Onset of CFRD. *J. Clin. Endocrinol. Metab.* 101 1274–1281. 26760176 10.1210/jc.2015-3730

[B70] Montgomery M. K. (2019). Mitochondrial Dysfunction and Diabetes: Is Mitochondrial Transfer a Friend or Foe? *Biology* 8:33. 10.3390/biology8020033 31083560 PMC6627584

[B71] Olefsky J. M. Glass C. K. (2010). Macrophages, inflammation, and insulin resistance. *Annu. Rev. Physiol.* 72 219–246. 10.1146/annurev-physiol-021909-135846 20148674

[B72] Park J. S. Svetkauskaite D. He Q. Kim J. Y. Strassheim D. Ishizaka A. (2004). Involvement of toll-like receptors 2 and 4 in cellular activation by high mobility group box 1 protein. *J. Biol. Chem.* 279 7370–7377. 10.1074/jbc.M306793200 14660645

[B73] Pyo J. O. Yoo S. M. Ahn H. H. Nah J. Hong S. H. Kam T. I. (2013). Overexpression of Atg5 in mice activates autophagy and extends lifespan. *Nat. Commun.* 4:2300. 10.1038/ncomms3300 23939249 PMC3753544

[B74] Raj S. Chandela V. Kumar A. Kesari K. K. Asthana S. Ruokolainen J. (2020). Molecular mechanisms of interplay between autophagy and metabolism in cancer. *Life Sci. Volume* 259 118–184. 10.1016/j.lfs.2020.118184 32763290

[B75] Raucci A. Di Maggio S. Scavello F. D’Ambrosio A. Bianchi M. E. Capogrossi M. C. (2019). The Janus face of HMGB1 in heart disease: a necessary update. *Cell. Mole. Life Sci.* 76 211–229. 10.1007/s00018-018-2930-9 30306212 PMC6339675

[B76] Reaven G. M. (2006). The metabolic syndrome: is this diagnosis necessary? *Am. J. Clin. Nutr.* 83 1237–1247. 10.1093/ajcn/83.6.1237 16762930

[B77] Ren J. Sowers J. R. Zhang Y. (2018). Metabolic Stress, Autophagy, and Cardiovascular Aging: from Pathophysiology to Therapeutics. *Trends Endocrinol. Metab.* 29 699–711. 10.1016/j.tem.2018.08.001 30145108 PMC6151141

[B78] Ren S. Y. Xu X. (2015). Role of Autophagy in Metabolic Syndrome-Associated Heart Disease. *Biochim. Biophys. Acta* 1852 225–231. 10.1016/j.bbadis.2014.04.029 24810277 PMC4221581

[B79] Rohde K. Rønningen T. La Cour Poulsen L. Keller M. Blüher M. (2020). Role of the DNA repair genes H2AX and HMGB1 in human fat distribution and lipid profiles. *BMJ Open Diab. Res. Care.* 8:e000831. 10.1136/bmjdrc-2019-000831 32114485 PMC7050360

[B80] Rubinsztein D. C. Mariño G. Kroemer G. (2011). Autophagy and aging. *Cell* 146 682–695. 10.1016/j.cell.2011.07.030 21884931

[B81] Ruderman N. B. Carling D. Prentki M. Cacicedo J. M. (2013). AMPK, insulin resistance, and the metabolic syndrome. *J. Clin. Invest.* 123 2764–2772. 10.1172/JCI67227 23863634 PMC3696539

[B82] Ryter S. W. Koo J. K. Choi A. M. (2014). Molecular regulation of autophagy and its implications for metabolic diseases. *Curr. Opin. Clin. Nutr. Metab. Care.* 7 329–337. 10.1097/MCO.0000000000000068 24848530 PMC4858436

[B83] Samidurai A. Kukreja R. C. Das A. (2018). Emerging Role of mTOR Signaling-Related miRNAs in Cardiovascular Diseases. *Oxidat. Med. Cell. Long.* 2018:6141902. 10.1155/2018/6141902 30305865 PMC6165581

[B84] Seidu S. Gillies C. Zaccardi F. Kunutsor S. K. Hartmann-Boyce J. Yates T. (2020). The impact of obesity on severe disease and mortality in people with SARS-CoV-2: A systematic review and meta-analysis. *Endocrinol. Diabetes Metab.* 14:e00176. 10.1002/edm2.176 32904932 PMC7460942

[B85] Shimizu T. Yamakuchi M. Biswas K. K. Aryal B. Yamada S. Hashiguchi T. (2016). HMGB1 is secreted by 3T3-L1 adipocytes through JNK signaling and the secretion is partially inhibited by adiponectin. *Obesity* 24 1913–1921. 10.1002/oby.21549 27430164

[B86] Sohrabi Y. Lagache S. M. M. Schnack L. Godfrey R. Kahles F. Bruemmer D. (2019). mTOR-Dependent Oxidative Stress Regulates oxLDL-Induced Trained Innate Immunity in Human Monocytes. *Front. Immunol.* 9:3155. 10.3389/fimmu.2018.03155 30723479 PMC6350618

[B87] Street M. E. (2020). HMGB1: A possible crucial Therapeutic Target for Covid-19? *Horm. Res. Paediatr.* 2020:000508291. 10.1159/000508291 32375153 PMC7251586

[B88] Takeshige K. Baba M. Tsuboi S. Noda T. Ohsumi Y. (1992). Autophagy in yeast demonstrated with proteinase-deficient mutants and conditions for its induction. *J. Cell Biol.* 119 301–311. 10.1083/jcb.119.2.301 1400575 PMC2289660

[B89] Tang D. Kang R. Cheh C. W. Livesey K. M. Liang X. Schapiro N. E. (2010b). HMGB1 release and redox regulates autophagy and apoptosis in cancer cells. *Oncogene* 29 5299–5310. 10.1038/onc.2010.261 20622903 PMC2945431

[B90] Tang D. Kang R. Livesey K. M. Cheh C. W. Farkas A. Loughran P. (2010a). Endogenous HMGB1 regulates autophagy. *J. Cell Biol.* 190 881–892. 10.1083/jcb.200911078 20819940 PMC2935581

[B91] Tang D. Kang R. Livesey K. M. Kroemer G. Billiar T. R. Van Houten B. (2011). High-mobility group box 1 is essential for mitochondrial quality control. *Cell Metab.* 13 701–711. 10.1016/j.cmet.2011.04.008 21641551 PMC3293110

[B92] Trim W. Turner J. E. Thompson D. (2018). Parallels in Immunometabolic Adipose Tissue Dysfunction with Ageing and Obesity. *Front. Immunol.* 9:169. 10.3389/fimmu.2018.00169 29479350 PMC5811473

[B93] Ueno T. Komatsu M. (2017). Autophagy in the liver: functions in health and disease. *Nat. Rev. Gastroenterol. Hepatol* 14 170–184. 10.1038/nrgastro.2016 28053338

[B94] Umahara T. Uchihara T. Hirao K. Shimizu S. Hashimoto T. Kohno M. (2020). Essential autophagic protein Beclin 1 localizes to atherosclerotic lesions of human carotid and major intracranial arteries. *J. Neurol. Sci.* 414:116836. 10.1016/j.jns.2020.116836 32344218

[B95] van Niekerk G. Davis T. Patterton H. G. Engelbrecht A. M. (2019). How Does Inflammation-Induced Hyperglycemia Cause Mitochondrial Dysfunction in Immune Cells? *Bioessays.* 41:e1800260. 10.1002/bies.201800260 30970156

[B96] Wang H. Qu H. Deng H. (2015). Plasma HMGB-1 levels in subjects with obesity and type 2 diabetes: a cross-sectional study in China. *PLoS One* 10:e0136564. 10.1371/journal.pone.0136564 26317615 PMC4552731

[B97] Wang L. Harris T. E. Roth R. A. Lawrence J. C. Jr. (2007). PRAS40 regulates mTORC1 kinase activity by functioning as a direct inhibitor of substrate binding. *J. Biol. Chem.* 282 20036–20044. 10.1074/jbc.M702376200 17510057

[B98] Wang Y. Zhong J. Zhang X. Liu Z. Yang Y. Gong Q. (2016). The Role of HMGB1 in the Pathogenesis of Type 2 Diabetes. *J. Diabetes Res.* 2016:2543268. 10.1155/2016/2543268 28101517 PMC5215175

[B99] Watanabe J. Kotani K. (2020). Metabolic Syndrome for Cardiovascular Disease Morbidity and Mortality Among General Japanese People: A Mini Review. *Vasc. Health Risk Manag.* 16 149–155. 10.2147/VHRM.S245829 32368073 PMC7182458

[B100] Weihe P. Weihrauch-Blûher S. (2019). Metabolic Syndrome in Children and Adolescents: Diagnostic criteria, therapeutic options and perspectives. *Curr. Obesity Rep.* 8 472–479. 10.1007/s13679-019-00357-x 31691175

[B101] Xu Y. Shena J. Ran Z. (2020). Emerging views of mitophagy in immunity and autoimmune diseases. *Autophagy* 16 3–17. 10.1080/15548627.2019.1603547 30951392 PMC6984455

[B102] Xue J. Suarez J. S. Minaai M. Li S. Gaudino G. Pass H. I. (2020). HMGB1 as a therapeutic target in disease. *J. Cell Physiol.* 26:30125. 10.1002/jcp.30125 33107103 PMC8104204

[B103] Yang H. Antoine D. J. Andersson U. Tracey K. J. (2013). The many faces of HMGB1: molecular structure-functional activity in inflammation, apoptosis, and chemotaxis. *J. Leukocyte Biol.* 93 865–873. 10.1189/jlb.1212662 23446148 PMC4051189

[B104] Yang L. Li P. Fu S. Calay E. S. Hotamisligil G. S. (2010). Defective Hepatic Autophagy in Obesity Promotes ER Stress and Causes Insulin Resistance. *Cell Metab.* 11 467–478. 10.1016/j.cmet.2010.04.005 20519119 PMC2881480

[B105] Yao Y. Guo D. Yang S. Lin Y. He L. Chen J. (2015). HMGB1 gene polymorphism is associated with hypertension in Han Chinese population. *Clin. Exp. Hypertens.* 37 166.171. 10.3109/10641963.2014.933963 25050807

[B106] Zhang J. Chen L. Wang F. Zou Y. Li J. Luo J. (2020). Extracellular HMGB1 exacerbates autoimmune progression and recurrence of type 1 diabetes by impairing regulatory T cell stability. *Diabetologia* 63 987–1001. 10.1007/s00125-020-05105-8 32072192 PMC7145789

[B107] Zhang J. Zhang L. Zhang S. Yu Q. Xiong F. Huang K. (2017). HMGB1, an innate alarmin, plays a critical role in chronic inflammation of adipose tissue in obesity. *Mol. Cell Endocrinol.* 15 103–111. 10.1016/j.mce.2017.06.012 28619625

[B109] Zhang Y. Sowers J. R. Ren J. (2018). Targeting autophagy in obesity: from pathophysiology to management. *Nat. Rev. Endocrinol* 14 356–376. 10.1038/s41574-018-0009-1 29686432

[B200] Zhang Y. Thery F. Wu N. C. Luhmann E. K. Dussurget O. Foecke M. (2019). The in vivo ISGylome links ISG15 to metabolic pathways and autophagy upon Listeria monocytogenes infection. *Nat. Commun.* 10. 10.1038/s41467-019-13393-x 31772204 PMC6879477

[B110] Zhu X. Messer J. S. Wang Y. Lin F. Cham C. M. Chang J. (2015). Cytosolic HMGB1 controls the cellular autophagy/apoptosis checkpoint during inflammation. *J. Clin. Investig.* 125 1098–1110. 10.1172/JCI76344 25642769 PMC4362239

